# High-throughput and direct measurement of androgen levels using turbulent flow chromatography liquid chromatography-triple quadrupole mass spectrometry (TFC-LC-TQMS) to discover chemicals that modulate dihydrotestosterone production in human prostate cancer cells

**DOI:** 10.1007/s10529-017-2480-5

**Published:** 2017-11-21

**Authors:** Kyungsu Kang, Lei Peng, Yu-Jin Jung, Joo Yeon Kim, Eun Ha Lee, Hee Ju Lee, Sang Min Kim, Sang Hyun Sung, Cheol-Ho Pan, Yongsoo Choi

**Affiliations:** 1 Systems Biotechnology Research Center, Korea Institute of Science and Technology, Gangneung, 25451 Republic of Korea; 2 Department of Biological Chemistry, Korea University of Science and Technology, Daejeon, 34113 Republic of Korea; 3 College of Pharmacy and Research, Institute of Pharmaceutical Sciences, Seoul National University, Seoul, 08826 Republic of Korea

**Keywords:** 5α-reductase inhibitor, Dihydrotestosterone, Fucoxanthin, Naringenin, Testosterone

## Abstract

**Objectives:**

To develop a high-throughput screening system to measure the conversion of testosterone to dihydrotestosterone (DHT) in cultured human prostate cancer cells using turbulent flow chromatography liquid chromatography-triple quadrupole mass spectrometry (TFC-LC-TQMS).

**Results:**

After optimizing the cell reaction system, this method demonstrated a screening capability of 103 samples, including 78 single compounds and 25 extracts, in less than 12 h without manual sample preparation. Consequently, fucoxanthin, phenethyl caffeate, and *Curcuma longa* L. extract were validated as bioactive chemicals that inhibited DHT production in cultured DU145 cells. In addition, naringenin boosted DHT production in DU145 cells.

**Conclusion:**

The method can facilitate the discovery of bioactive chemicals that modulate the DHT production, and four phytochemicals are potential candidates of nutraceuticals to adjust DHT levels in male hormonal dysfunction.

**Electronic supplementary material:**

The online version of this article (10.1007/s10529-017-2480-5) contains supplementary material, which is available to authorized users.

## Introduction

The male androgen hormone, testosterone, is converted to dihydrotestosterone (DHT) by 5α-reductases. Since DHT has a much higher binding affinity to the androgen receptor than testosterone, DHT is an active metabolite of testosterone. It is involved in several diseases in older males, including benign prostatic hyperplasia and androgenic alopecia. Clinical 5α-reductase inhibitors, which are finasteride and dutasteride, can potently decrease the serum and prostatic DHT and are used to treat male patients with these diseases (Azzouni et al. 2012; Azzouni and Mohler 2012). However, finasteride and dutasteride exert various adverse effects such as sexual dysfunction, infertility, and depression (Yim et al. 2014). Thus, investigations to discover new 5α-reductase inhibitors with weaker adverse effects are ongoing (Hiipakka et al. 2002).

Traditionally, two radio-label methods, which involve [^3^H]- and [^14^C]-testosterone, are used for inhibitory screening assays of the enzyme reaction. In these methods, each type of radio-labeled testosterone is used as a substrate for the enzyme reaction with candidate inhibitors, and the resulting radio-labeled DHT is measured using scintillation spectrometry and thin-layer chromatography (TLC) (Hiipakka et al. 2002; Kim and Ma 2009). However, these methods require a large amount of radio-labeled testosterone (in the range of a few µmol), which is much higher than the physiologically relevant concentration of testosterone (Hiipakka et al. 2002; Kim and Ma 2009). Alternatively, rat microsomal suspensions (Suphrom et al. 2012) or cell homogenates extracted from androgen-dependent cells that express the enzymes (Srivilai et al. 2016) are used for enzyme assays with high testosterone concentrations. After the enzyme reaction, the levels of testosterone and DHT in the reaction solution are determined using HPLC–UV or are chemically derivatized for LC–MS analysis. However, this method requires labor-intensive sample preparation for both chemical derivatization and sample extraction (Srivilai et al. 2016).

Since 5α-reductases are membrane-bound enzymes with a high content of hydrophobic amino acids (Azzouni et al. 2012), it is extremely difficult to express recombinant membrane proteins in an active form using *Escherichia coli* (Angius et al. 2016). An in vitro biochemical assay using the recombinant enzyme is not applicable. Therefore, a cell-based high-throughput screening system with minimal sample preparation and maximal sensitivity should be developed to efficiently screen 5α-reductase inhibitors in complex biological matrices.

To determine androgen metabolites levels in biological matrices, MS analysis is often used (Srivilai et al. 2016; Zang et al. 2017). However, this requires chemical derivatization of androgen metabolites or the radio-labeled substrates, which make them difficult for the high-throughput screening. In the present study, we develop a high-throughput screening system based on the direct and rapid determination of DHT produced from cultured prostate cells using TFC-LC-TQMS without labor-intensive manual sample preparation. We have also discovered bioactive chemicals that modulate the DHT production for the potential candidates of nutraceuticals associated with male hormonal dysfunctions.

## Materials and methods

### Reagents

Testosterone, and dihydrotestosterone (DHT) were from Sigma-Aldrich. The internal standard of 2-(1-(4-chlorobenzoyl)-5-methoxy-2-methyl-1H-indol-3-yl)acetic acid was provided by Seoul National University, College of Pharmacy.

### Cell culture

CWR-22Rv1 (22Rv1), LNCaP, DU145, and PC-3 human prostate cancer cells were purchased from ATCC.

### Chemical treatment of prostate cancer cells

To select the appropriate cell lines for the assay, 22Rv1, LNCaP, DU145, and PC-3 cells (3 × 10^5^ cells per well) were seeded in a 96-well plate and incubated for 24 h. Then, the cells were treated with vehicle control (0.2% DMSO), testosterone, or testosterone plus finasteride for 6–96 h. For the screening, DU145 cells (10^4^ cells per well) were seeded in a 96-well plate and incubated for 24 h. The cells were incubated with 300 μl fresh RPMI medium supplemented with the vehicle control, testosterone alone, or testosterone and tested chemicals for 48 h. Then, 200 μl supernatant medium was transferred to a new tube and stored at − 20 °C for further MS analysis. After sampling, the cell viability of DU145 cells was determined by measuring the mitochondrial dehydrogenase activity as previously described (Kang et al. 2014).

### TFC-LC-TQMS analysis

The details on the mechanical connections of TFC with LC–MS/MS were described in the previous study (Shin et al. 2016). First, 20 μl aliquots of cell supernatant that contained 50 nM of internal standard, 2-(1-(4-chlorobenzoyl)-5-methoxy-2-methyl-1H-indol-3-yl)acetic acid were directly injected into the TFC column (Cyclone P column; 0.5 × 50 mm) using an Agilent 1260/1290 dual UPLC system. We selected this chemical as the internal standard because it has similar retention time to DHT, which is a key metabolite for the screening. The TFC solvent system consisted of water/acetonitrile (95:5, v/v with 0.1% formic acid) as Solvent A and acetonitrile as Solvent B. After the sample loading, 40% Solvent B was started at 2 ml/min for 0.5 min; then, the TFC column was reversely connected with a Waters XTerra MS C_18_ analytical column (2.1 × 100 mm, 3.5 µm) by a switching valve. Testosterone and DHT were chromatographically separated in 65–90% Solvent B at 0.25 ml/min for 4 min. Then, the switching valve was turned to separate the TFC and analytical column for re-equilibrium, where 65% Solvent B was used for 3.5 min. The following condition was used for the TFC column washing and re-equilibrium: 100% of Solvent B at 2 ml/min for 2 min and subsequently 40% of Solvent B at 2 ml/min for 1.5 min.

Triple quadrupole mass spectrometry (TQMS; AB SCIEX API 4000 QTRAP, Foster City, CA, USA) was interfaced to an Agilent 1260/1290 dual UPLC system (Palo Alto, CA, USA). The solvent system (at 0.25 ml/min) consisted of water/acetonitrile (95:5, v/v with 0.1% formic acid) as Solvent A and acetonitrile/water (95:5, v/v containing 0.1% formic acid) as Solvent B. The column was maintained at room temperature. Positive-ion electrospray tandem mass spectra were recorded, and the obtained results were processed with the Analyst 1.6.2 software. The ion spray voltage was set to 5500 V, and the probe was set to 400 °C. N_2_ was used as the curtain gas, GS1, and GS2 at 30, 35, and 40, respectively. The selected reaction monitoring (SRM) parameters of the analytes are presented in Table 1. The two SRM transitions for testosterone, DHT, and internal standard were used to enhance the selectivity of the detection signal in the culture medium. The SRM transitions for testosterone and dihydrotestosterone were reported (Upreti et al. 2015).

**Table 1:** The selected reaction monitoring (SRM) parameters of testosterone, dihydrotestosterone (DHT), and internal standard

Compound	SRM transition (*m/z*)	DP (eV)	EP (eV)	CE (eV)	CXP (eV)
Testosterone	289/97	96	10	33	16
	289/109	96	10	37	18
Dihydrotestosterone	291/255	101	10	23	14
	291/77	101	10	93	12
Internal standard	358/139	71	10	25	10
	358/111	71	10	73	18

Internal standard: 2-(1-(4-chlorobenzoyl)-5-methoxy-2-methyl-1H-indol-3-yl)acetic acid

*DP* declustering potential, *EP* entrance potential, *CXP* collision cell exit potential

### Statistical analysis

The data are expressed as the mean ± standard deviation (SD). Statistical analyses were performed using a one-way analysis of variance (ANOVA) and subsequently Dunnett’s multiple comparison test or Tukey’s multiple comparison test using the GraphPad Prism 5 software (GraphPad Software, Inc., La Jolla, CA, USA).

## Results

### Cell-based reaction coupled with TFC LC–MS/MS

**Fig. 1:**
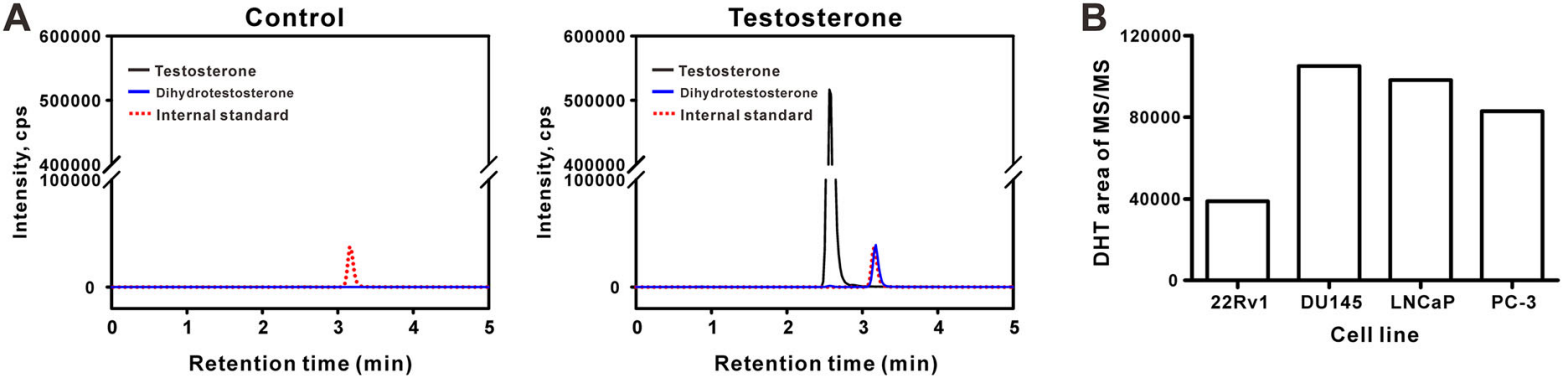
Conversion of testosterone to dihydrotestosterone (DHT) in human prostate cancer cells. **a** Representative TFC LC-MS/MS chromatogram obtained from the cell culture medium of DU145 cells. DU145 cells were treated with DMSO (0.2%) or testosterone (100 ng/ml) for 48 h. **b** Relative abundance of DHT in various human prostate cancer cells. 22Rv1, DU145, LNCaP, and PC-3 cells were treated with testosterone (200 ng/ml) for 48 h

**Fig. 2:**
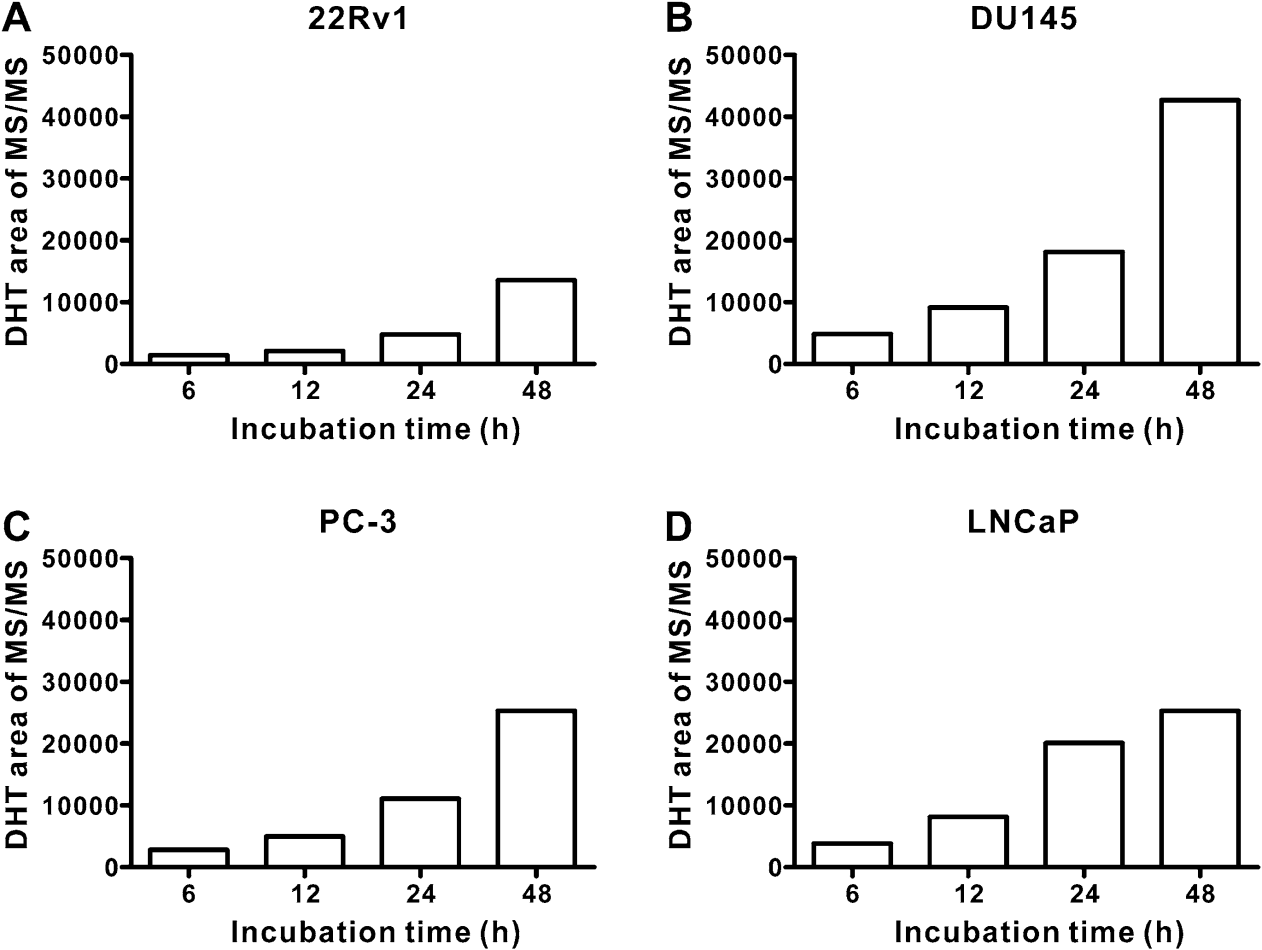
Time-dependent conversion of testosterone to DHT in various human prostate cancer cells. 22Rv1, DU145, LNCaP, and PC-3 cells were treated with testosterone (100 ng/ml) for 6, 12, 24, and 48 h. The relative abundance of DHT in various human prostate cancer cells: **a** 22Rv1, **b** DU145, **c** PC-3, and **d** LNCaP cells

First, we evaluated 5α-reductase activity by measuring the production of DHT in cultured human prostate cancer cells treated with testosterone using the TFC LC–MS/MS method. For this evaluation, the SRM parameters for testosterone and DHT were optimized as shown in Table 1. Four different prostate cancer cell lines (22Rv1 and LNCaP cells, which are androgen receptor-positive cells; DU145 and PC-3 cells, which are androgen receptor-negative cells) were used to determine the best cell line for the screening system. As shown in Fig. 1a, the TFC LC–MS/MS method, whose detection limits of testosterone and DHT were 0.75 and 2.25 ng/ml, respectively, did not show any signal in the control where DU145 cells were treated with DMSO. However, the TFC LC–MS/MS method showed two prominent signals of testosterone and DHT at 2.65 min and 3.3 min, respectively, when the cells were treated with 100 ng testosterone/ml for 48 h (Fig. 1a). The apparent conversion of testosterone to DHT was also observed in all other cell lines and could be monitored by TFC LC–MS/MS (Fig. 1b). This result demonstrates that the TFC LC–MS/MS method is suitable to discover bioactive chemicals that modulate the DHT production in cell reaction systems. To investigate the time-dependent conversion of testosterone to DHT in various human prostate cancer cells, 4 different prostate cancer cells were treated with testosterone for 6, 12, 24, and 48 h. All human prostate cancer cells showed a time-dependent increase in DHT production and the highest DHT production at 48 h regardless of cell type (i.e., androgen receptor-positive or negative) (Fig. 2). Among four different prostate cell lines, DU145 had the highest enzyme activity, and there was an 8.8-fold increase in DHT production at 6–48 h in the cell. Thus, DU145 cell with 48 h of testosterone treatment was selected as a cell reaction condition to discover and evaluate the bioactive modulators of the DHT production.

### Method validation and primary screening

**Fig. 3:**
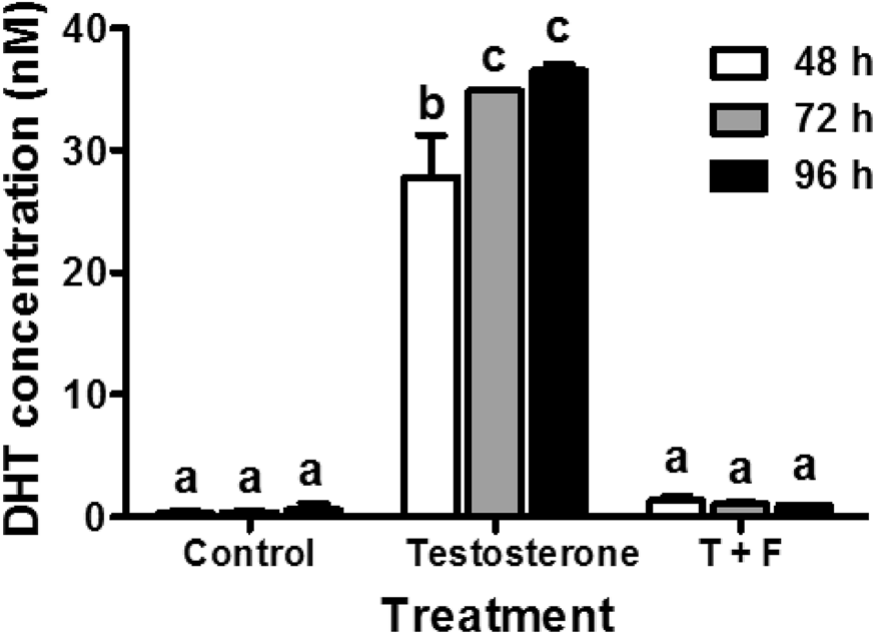
Effects of finasteride on the conversion of testosterone to DHT in DU145 cells. The DU145 cells were treated with testosterone (T, 100 ng/ml) and finasteride (F, 10 μM) for 48, 72, and 96 h. The values represent the mean ± SD from duplicate experiments. The bars with different letters are significantly different (*p* < 0.01, ANOVA, Tukey’s multiple comparison test)

After optimizing the cell reaction system, the TFC LC–MS/MS method was used to verify that a clinical 5α-reductase inhibitor (finasteride) could inhibit the production of DHT in DU145 cells. As expected, the inhibitor completely abrogated the conversion of testosterone to DHT (Fig. 3). The determined final concentration of DHT for the 72- and 96-h treatment was only 1.26- and 1.32-fold higher than that of the 48-h treatment. Thus, the 48-h treatment time for the screening system is evidently sufficient to monitor the 5α-reductase activity in DU145 cells.

**Fig. 4:**
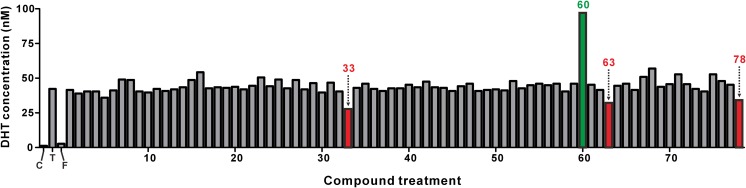
Screening of the compounds that modulate the DHT production in DU145 cells. The DU145 cells were treated with vehicle control, testosterone (T, 100 ng/ml), testosterone (100 ng/ml) plus finasteride (F, 10 μM), and testosterone (100 ng/ml) plus various compounds (20 μM) for 48 h. The sample numbers of compounds that decreased the DHT concentration to below 35 nM are indicated by the red color. Naringenin (Compound #60) increased the DHT concentration to 97 nM, as indicated by the green color

**Fig. 5:**
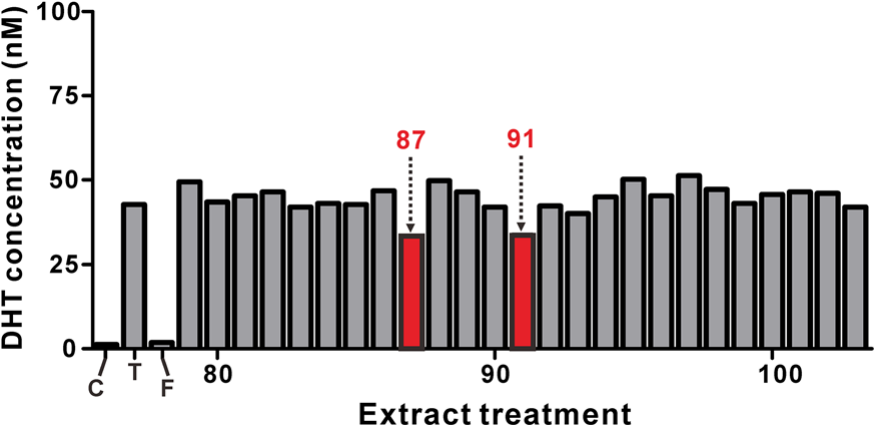
Screening of extracts to modulate the DHT production in DU145 cells. The DU145 cells were treated with vehicle control, testosterone (T, 100 ng/ml), testosterone (100 ng/ml) plus finasteride (F, 10 μM), and testosterone (100 ng/ml) plus various extracts (20 μg/ml) for 48 h. The sample numbers of extracts that decreased the DHT concentration to below 35 nM are indicated by the red color

Then, we performed the primary screening of 78 single compounds and 25 extracts using the TFC LC–MS/MS method. Details on the names of the compounds and extracts are summarized in Supplementary Tables 1 and 2, respectively. In our cell-based screening system, the primary screening hits were determined by a threshold of more than 18% change of the DHT production at 20 μM phytochemical or 20 μg/ml of extract. With this criterion, four compounds and two plant extracts were found to be primary hits that modulated the DHT levels (Figs. 4, 5). Notably, naringenin (#60 in Supplementary Table 1) increased the DHT production more than two-fold compared to the testosterone-only treatment (Fig. 4).

### Secondary screening and hit evaluations

**Fig. 6:**
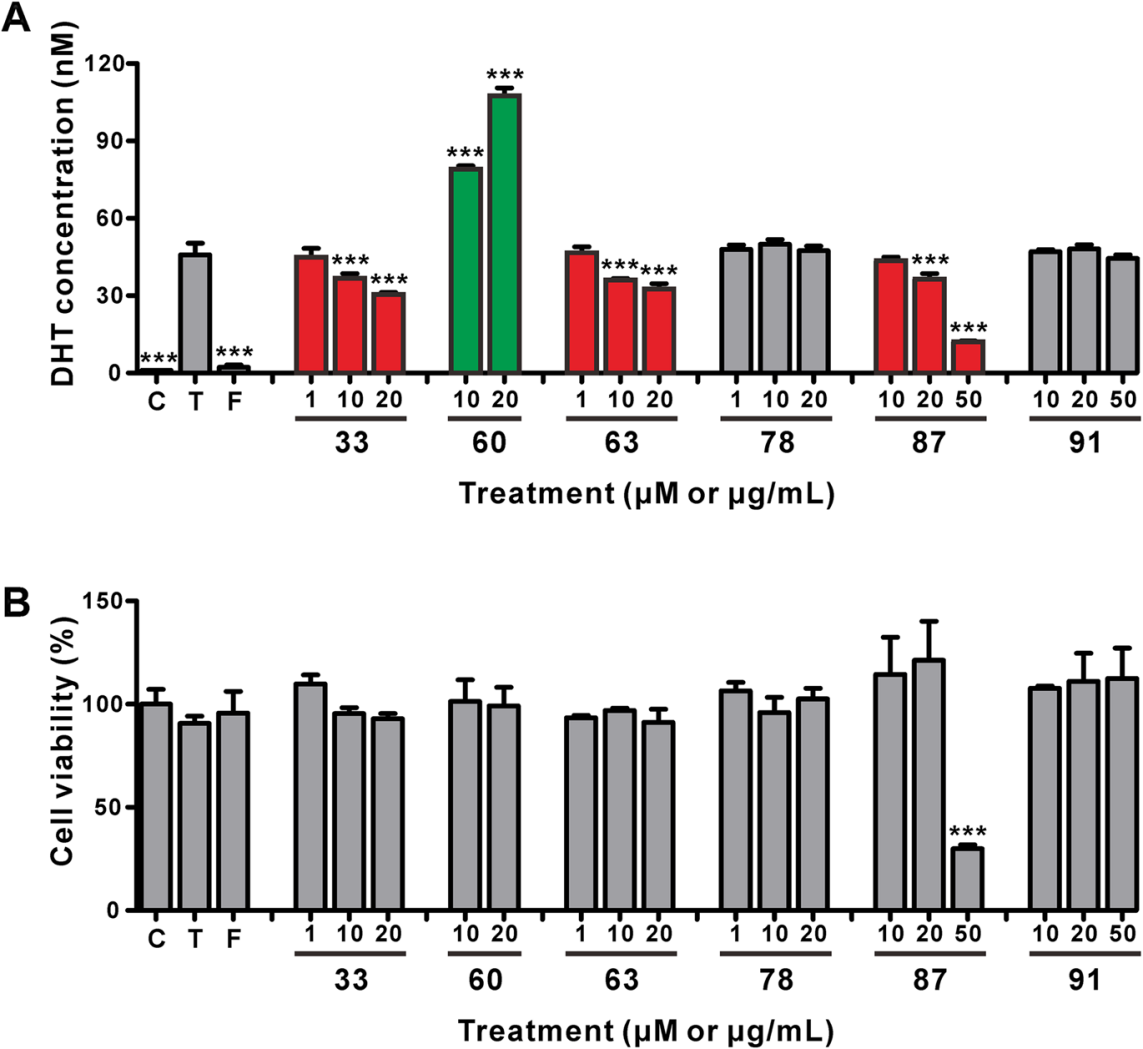
Effects of the candidate compounds and extracts on the DHT production and cell viability in DU145 cells. The DU145 cells were treated with vehicle control, testosterone (T, 100 ng/ml), testosterone (100 ng/ml) plus finasteride (F, 10 μM), and testosterone (100 ng/ml) plus various compounds (fucoxanthin, compound # 33, 1–20 μM; naringenin, compound # 60, 10–20 μM; phenethyl caffeate, compound # 63, 1–20 μM; tigloylgomisin H, compound # 78, 1–20 μM) or extracts (*Curcuma longa* extract, extract # 87, 10–50 μg/ml; *Hydrangea serrata* ethanol extract, extract # 91, 10–50 μg/ml) for 48 h. **a** DHT production in DU145 cells. The values represent the mean ± SD from triplicate experiments. ***p < 0.001 for significant differences from the testosterone single treatment (ANOVA, Dunnett’s multiple comparison test). **b** Cell viability of DU145 cells that were treated with various chemicals for 48 h. The values represent the mean ± SD from triplicate experiments. ***p < 0.001 for significant differences from the vehicle control (ANOVA, Dunnett’s multiple comparison test)

Based on the primary hit results, subsequent dose-dependency experiments were performed to evaluate the activity of the primary hits at 2–3 different concentrations. The results in Fig. 6a show that two compounds (#33, fucoxanthin and #63, phenethyl caffeate) and one plant extract (#87, *Curcuma longa* extract) were discovered as phytochemicals that inhibited the DHT production. Additionally, we confirmed that naringenin (#60) substantially increased the DHT production in DU145 cells in a dose-dependent manner (Fig. 6a). We also checked these phytochemicals at concentrations below 20 μM for the compounds and below 20 μg/ml for the extracts, which we found to not affect the cell viability, and their DHT modulating activity did not originate from different cell viabilities (Fig. 6b).

## Discussion

Here, we have established a high-throughput method to determine the DHT production in cultured DU145 cells using the TFC LC–MS/MS. Our method is rapid, convenient, sensitive, reproducible, and safe compared to other previous methods using TLC or HPLC, which require a high amount of radio-labeled testosterone or chemical derivatization because of their lower sensitivity (Hiipakka et al. 2002; Kim and Ma 2009; Srivilai et al. 2016). For the secondary screening and hit evaluation, we performed two independent experiments and observed consistent results. The TFC LC–MS/MS method based on a cell-based screening system successfully discovered bioactive phytochemicals that inhibit the conversion of testosterone to DHT, although their inhibitory potencies on the DHT production were weaker than the therapeutic positive, finasteride. Among the discovered phytochemicals, phenethyl caffeate and *Curcuma longa* extract were reported as 5α-reductase inhibitors in previous studies (Hiipakka et al. 2002; Srivilai et al. 2016). Fucoxanthin was first reported as an inhibitor of the DHT production in the current study. Fucoxanthin is a major compound present in edible seaweed *Eisenia bicyclis* and microalgae *Phaeodactylum tricornutum* (Kim et al. 2012).

Interestingly, we also discovered that naringenin increased the production of DHT in DU145 cells. The detailed mechanisms of naringenin, which boosts the DHT production in prostate cells, should be further studied. According to the steroid hormone biosynthesis in the KEGG pathway database, testosterone is a precursor of few metabolites such as 5α-DHT, 5β-DHT, testosterone glucuronide, and estradiol, which are produced by 5α-reductase, 5β-reductase, glucuronosyltransferase, and aromatase, respectively. According to the previous study, naringenin inhibits the aromatase activity (Edmunds et al. 2005). Therefore, the inhibitory action of naringenin on the aromatase enzyme may increase the metabolite pathway flux of the DHT production by 5α-reductase.

Initially, we expected that the proliferation of prostate cancer cells would be differently induced depending on the DHT levels and could be used for the inhibitory screening of 5α-reductase, since DHT has a 10-fold higher potency in inducing androgen signaling compared to testosterone (Azzouni et al. 2012; Azzouni and Mohler 2012). Based on this speculation, we measured the cell proliferation of two androgen receptor-positive prostate cancer cells; LNCaP and 22Rv1. The cells were treated for 4–12 days with only testosterone (1 or 10 nM) or testosterone with test compounds (finasteride, fucoxanthin, and naringenin). Contrary to our expectations, the cell proliferation did not depend on these DHT modulators (data not shown). For example, we could not observe an additive cell proliferation by the treatment of naringenin, which is a stimulator of the DHT production, compared to the testosterone-only treatment. This result may occur because the stimulation potencies of testosterone and DHT on the cell proliferation of prostate cancer cells are not dramatically different in vitro, according to our preliminary experiments (data not shown). Similar results were observed in previous reports, where the cell proliferation did not vary when LNCaP prostate cancer cells were treated with 0.1–1000 nM testosterone or DHT (Arnold et al. 2005).

These findings confirm that our high-throughput method to discover DHT modulators from chemical library using TFC LC–MS/MS is invaluable because neither the cell-based proliferation assay nor the biochemical assay using recombinant proteins are applicable for this purpose.

**In summary**, we have developed a cell-based high-throughput screening system to discover chemical modulators of the DHT production using the TFC LC–MS/MS method. The developed method demonstrates a screening capability of 103 samples in less than 12 h without manual sample preparation. This method successfully discovered four potential phytochemicals that modulated the DHT production in human prostate cancer cells. These phytochemicals can be useful for those who need to normalize the androgen levels by boosting or inhibiting the DHT production. However, further in-depth studies including preclinical efficacy and toxicological evaluation are necessary to develop them as androgen-modulatory nutraceuticals.

## Electronic supplementary material

Below is the link to the electronic supplementary material.

Supplementary material 1 (PDF 147 kb)
