# Supplementary material for: High-throughput and direct measurement of androgen levels using turbulent flow chromatography liquid chromatography-triple quadrupole mass spectrometry (TFC-LC-TQMS) to discover chemicals that modulate dihydrotestosterone production in human prostate cancer cells
Source: Biotechnol Lett. 2017 Nov 21;40(2):263–70. doi: 10.1007/s10529-017-2480-5 (PMC5813082; doi:10.1007/s10529-017-2480-5)
Supplement: Supplementary file 1 — Supplementary material 1 (PDF 147 kb) [file 10529_2017_2480_MOESM1_ESM.pdf]

Supplementary Table 1 Compounds tested for modulatory activity on the DHT production in DU145 cells

Number	Compound name	Number	Compound name
1	[10]-Gingerol	40	Glycyrrhizin
2	[4]-Gingerol	41	Gomisin J
3	[6]-Gingerol	42	Gomisin N
4	[6]-Shogaol	43	Hexahydrocurcumin
5	[8]-Gingerol	44	Hyperin
6	14-Episinomenine	45	Isoliquiritigenin
7	3,3'-Diindolylmethane	46	Isoliquiritin
8	4-Hydroxycoumarin	47	Isoliquiritin apioside
9	8-Demethoxyrunanine	48	Isoquercitrin
10	Acacetin	49	Isoquercitrin
11	Acetylsalicylic acid	50	Kaempferin
12	Acutumidine	51	Kaempferol
13	Acutumine	52	Lanosterol
14	Allyl sulfide	53	Licochalcone A
15	Amentoflavone	54	Licoflavonol
16	Angeloylgomisin H	55	Liquiritigenin
17	Apigenin	56	Liquiritin
18	Baicalein	57	Liquiritin apioside
19	Bisdemethoxycurcumin	58	Magnoflorine
20	β-Carotene	59	Myricitrin
21	Catechin	60	Naringenin
22	Chloramphenicol	61	Oleanolic acid
23	Chlorogenic acid hemihydrate	62	Ononin
24	Curcumin	63	Phenethyl Caffeate
25	Daidzein	64	Pycnogenol
26	D-Arabinose	65	Quercetin
27	Dihydroquercetin	66	Quercitrin
28	Epigallocatechin	67	Schisandrin A
29	Ergosterol peroxide	68	Schisandrin B
30	Etoposide	69	Schisandrin C
31	Fisetin	70	Schisandrol A
32	Formononetin	71	Schisandrol B
33	Fucoxanthin	72	Semilicoisoflavone B
34	Fucoxanthinol	73	Silymarin
35	Gallic acid	74	Sinomenine
36	Genistein	75	Tectoridin
37	Glabrol	76	*tert*-Butylhydroquinone
38	Glabrone	77	Tetracycline hydrochloride
39	Glycyrrhetinic acid	78	Tigloylgomisin H

Supplementary Table 2 Extracts tested for modulatory activity on the DHT production in DU145 cells

Number	Extract name	Extraction solvent	Reference
79	Antioxidant-enriched fraction from purple potato		(Kim et al. 2017)
80	Antioxidant-enriched fraction from red potato		(Kim et al. 2017)
81	*Auricularia auricula-judae* (Bull.) Wettst.	Ethanol	
82	*Auricularia auricula-judae* (Bull.) Wettst.	Water	
83	*Chloranthus japonicas* Siebold	Methanol	
84	*Citrus aurantium* L.	Methanol	
85	*Codonopsis lanceolate* (Siebold & Zucc.) Trautv.	Ethanol	
86	*Coix lacrymajobi* var. mayuen (Rom.Caill.) Stapf	Ethanol	
87	*Curcuma longa* L.	Ethanol	
88	*Dendropanax morbifera* H.Lev.	Water	
89	*Forsythia koreana* (Rehder) Nakai	Methanol	
90	*Hericium erinaceus*	Ethanol	
91	*Hydrangea serrata* Seringe	Ethanol	
92	*Hydrangea serrata* Seringe	Water	
93	*Inonotus obliquus* (persoon) Pilat	Ethanol	
94	*Platycodon grandifloras* (Jacq.) A.DC.	Ethanol	
95	Propolis	Water	
96	Red yeast rice	Water	
97	Red yeast rice	Ethanol	
98	*Selaginella tamariscina* (P. Beauv.) Spring	Ethanol	
99	*Selaginella tamariscina* (P. Beauv.) Spring	Water	
100	*Selaginella tamariscina* (P. Beauv.) Spring	Methanol	
101	*Senna tora* (L.) Roxb.	Ethanol	
102	*Thuja orientalis* L.	Methanol	
103	*Thuja orientalis* L.	Ethanol	

Article title: High-throughput and direct measurement of androgen levels using turbulent flow chromatography liquid chromatography-triple quadrupole mass spectrometry (TFC-LC-TQMS) discovered chemicals that modulate the dihydrotestosterone production in human prostate cancer cells
Journal name: Biotechnology Letters
Author names: Kyungsu Kang, Lei Peng, Yu-Jin Jung, Joo Yeon Kim, Eun Ha Lee, Hee Ju Lee, Sang Min Kim, Sang Hyun Sung, Cheol-Ho Pan, Yongsoo Choi

<Reference>

Kim DH et al. (2017) The protective effect of antioxidant enriched fractions from colored potatoes against hepatotoxic oxidative stress in cultured hepatocytes and mice. J Food Biochem 41:e12315
